# Sequential Extraction of Potentially Toxic Metals: Alteration of Method for Cu-Ni Polluted Peat Soil of Industrial Barren

**DOI:** 10.3390/toxics8020039

**Published:** 2020-06-02

**Authors:** Marina V. Slukovskaya, Irina P. Kremenetskaya, Svetlana V. Drogobuzhskaya, Andrey I. Novikov

**Affiliations:** 1 Tananaev Institute of Chemistry and Technology of Rare Elements and Mineral Raw Materials, Kola Science Centre, Russian Academy of Sciences, Academic Campus 26a, 184209 Apatity, Russia; i.kremenetskaia@ksc.ru (I.P.K.); s.drogobuzhskaia@ksc.ru (S.V.D.); a.novikov@ksc.ru (A.I.N.); 2 Laboratory of Nature-inspired Technologies and Environmental Safety of the Arctic, Kola Science Centre, Russian Academy of Sciences, Fersmana str. 14, 184209 Apatity, Russia

**Keywords:** copper, nickel, metal mobility, ammonium acetate buffer

## Abstract

An evaluation of fraction composition and transformation of metal compounds emitted by metal ore processing enterprises and accumulated in soils is crucial for assessing the environmental risks of pollution and ecosystem benefit of remediation. The aim of this study was to develop a suitable sequential fractional procedure for metal pollutants for the peat soils matrix in the impact zone of a Cu-Ni smelter. Three experiment series were performed: (a) the study of the effect of ammonium acetate buffer pH in the range of 3.7–7.8 on the soil metal extraction; (b) the study of the effect of additional volume and frequency of soil treatment with solutions on the content of water-soluble, ammonium acetate extractable, and 0.1 N HNO_3_ extractable fractions; and, (c) the determination of the metal fraction composition in the modified technique. Soil treatment with ammonium acetate buffer with a pH range of 4.5–5.5 was the most appropriate for the determination of mobile compounds of Cu and other metals in highly polluted peat soil. Triple soil treatment with water and ammonium acetate is necessary for the complete extraction of the water-soluble and exchangeable fractions, respectively. Additionally, we propose a procedure of full extraction of the exchangeable metal fraction from peat soils while using single treatment with 0.1 N HNO_3_. This scheme allows evaluating geochemical mobility of metals and current environmental harm of polluted soils with a high content of organic matter.

## 1. Introduction

This study of the content and transformation of metal compounds in soils polluted due to the activities of enterprises engaged in processing metal ores is an important task that allows for us to assess the environmental risks of industrial pollution and predict the effect of soil pollution on other living and non-living components of ecosystems [1,2]. The content of mobile compounds of metal pollutants is the main qualitative indicator of the effectiveness of geochemical barriers in the field remediation experiments [3].

A study of only the gross metal content is not sufficient for the assessment of metal behavior in the soil [1,4]. The presence of metal compounds, differing in both mobility and soil fixation mechanisms, determines the degree of their environmental hazard and requires a detailed study [5,6,7]. The use of specific extractants allow us to divide different groups of metal ions, which are called the metal fractions. The sequential extraction of metal compounds from soils, including the subsequent treatment of the same soil sample with selected solutions, make obtaining a complete picture of the distribution of the metal fraction possible [4,6,7].

The most of sequential extraction procedures separate the following metal fractions: (a) water-soluble; (b) exchangeable; (c) reducible bound to Fe/Mn oxides/hydroxides; (d) oxidizable bound to organic matter (OM) and sulfides; and, (e) residual—compounds firmly bound in the crystal lattices of stable minerals [7,8,9,10] (Table 1).

Water-soluble and exchangeable fractions are the most mobile and bioavailable forms of metal compounds in soils [8]. Metal fractions that are associated with OM and Fe/Mn oxides/hydroxides include compounds that are strongly bound to their carrier phases, actually do not migrate, but are potentially capable of migration under the changing redox conditions. The metal compounds in the residual fraction are only possible in strong acids [15].

It is impossible to achieve 100% selectivity by sequential extraction due to a large number of metal compounds in soil, as their properties can both vary significantly or be similar to each other. Therefore, the choice of extraction method is a compromise between a stronger, but less selective, and less strong, but more selective reagents [7,16,17].

Ammonium acetate buffer (AAB) is an extracting reagent of complex action, demonstrating various types of interaction with the soil [7,8,18]. The metal fraction extracted by AAB is referred to as the exchangeable fraction, which is actually mobile. The metal content in the exchangeable fraction often evaluates the toxicological status of contaminated soils [6,12,19]. The effect of AAB on different metal compounds varies considerably, and this poses difficulties in the comparison of metal mobility in soils. The buffer solution affects soil through the mechanisms of the complexation and the ion exchange. Ladonin [9] demonstrated that the more stable are acetate complexes of chemical elements, the more significant contribution of complexation into to extraction of metals from the soil. Accordingly, AAB extracts more metals than ammonium and acetic acid solutions separately. This feature ensures that not only mobile, but also weakly sorbed metal compounds, can be extracted during the soil treatment with AAB [14].

For highly polluted soils, the extraction procedure might differ from the conventional one. Studies of the changes of the number of treatments with extractant, its volume and pH, and duration and speed of shaking with the solution were reported [16,19,20].

Besides the exchangeable fraction, the water-soluble fraction of pollutants also should be analyzed as the most mobile metal fraction [13]. For industrially polluted peat soil, we also propose the determination of the metal amount extracted by 0.1 N HNO_3_, because this acid can extract more strongly bound metal compounds than AAB [2,21]. The use of such a sequence of extractants as water, AAB, and 0.1 N HNO_3_ allowed us to describe the sorption of both Cu and Ni by vermiculite and lizardite [22].

The activity of the copper-nickel enterprises in the Murmansk region of Russia led to the hard pollution of ecosystems and the formation of industrial barrens [23]. Analysis of soil polluted by emissions of non-ferrous smelters is not a routine due to the extremely high levels of contamination of Cu and Ni that belong to the potentially toxic metals (PTM) [24]. Over the decade, field experiments have been carried out on these territories in order to remedy the highly polluted and degraded soil using mining wastes of various mineral compositions [25,26]. In particular, the experiment conducted on peat soil, because this type of soil is dominating in depressions. The high pollution of the organic soils is promoted by its high acidity and the ability of OM to accumulate metals [24,27]. The determination of metal content in mobile geochemical fractions is the important task for the monitoring of the current state of soil metal pollution and remediation. Most of the standard analytical procedures are developed for soils with lower content of mobile metal compounds or soils artificially spiked by metal salts. Therefore, the development and justifying the analytical method for the most mobile (and, thereby, toxic) metal fractions in extremely polluted peat soil is needed.

This research aimed to develop the optimal method of the analysis of mobile metal form in extremely polluted peat soil in the impact zone of the copper-nickel plant. The tasks were: (1) the determination of the pH of extractant for the analysis of the exchangeable fraction; (2) the modifying of the standard procedure (increase in the volume of extractant or the number of soil treatments by extractant) providing a high level of extraction of mobile metal compounds (water-soluble and exchangeable fractions); and, (3) the study of 0.1 N nitric acid as the alternative reagent for the full extraction of mobile metal compounds from the peat soil.

## 2. Materials and Methods

### 2.1. Soil Sampling and Preparation

The study site was located at the industrial barren in 0.7 km from the Cu/Ni smelter of the Kola Mining and Metallurgical Company (Monchegorsk site, 67°55’7′ N, 32°51’5′ E) in the Murmansk Region of Russia. Peat eutrophic soil (Eutric Histosol) was sampled from the depth 0–5 cm, as this layer is the most contaminated. The soil was collected from 10 equidistant points on the total area of 400 m^2^. The mixed soil sample was air-dried at a temperature of 20 ± 2 °C and sieved (mesh size of 1.25 mm); the magnetic fraction was removed from the soil.

### 2.2. Methods of Analysis

Potentiometric measurements (with the water ratio of 1:25) determined the values of pH and Eh in soil samples [28].

The total content of chemical elements in peat soil was determined after autoclave microwave decomposition in the SW4 system in the DAK100 autoclaves (Berghof, Eningen, Germany) while using a mixture of HF and HNO_3_ concentrated acids.

Three experiment series were conducted in order to study the influence of the extraction conditions on the metal content in geochemical fractions.

In the first series, the effect of the pH values of AAB on the extraction of chemical elements was studied. In the first step, a water-soluble fraction was removed from the peat soil by treatment with distilled water (2 g soil per 50 mL of water) to eliminate the contribution of the most mobile fraction. Subsequently, the samples were treated with a 50 mL AAB solution. Nine AAB solutions with pH values of 3.7–7.8 were prepared by mixing 1 M NH_4_CH_3_COO and 1 M CHCOOH at different ratios [27]. After 6 h extraction, the solutions were filtered through a membrane filter, and the metal concentrations were determined.

In the second series, different treatments of soil by extractants (distilled water, AAB at pH 4.65, 0.1 N HNO_3_) were investigated: (1) multiple sequential treatment by water and AAB (one-, two-, and three-fold; with soil: solution ratio 2:50); (2) the two-fold increase of extractant volume (water and AAB; 2 g soil per 100 mL solution); and, (3) one-fold soil treatment by 0.1 N HNO_3_.

In the third series, the influence of extraction conditions of the water-soluble and exchangeable fractions (second series, actually mobile form) on the distribution of chemical elements in the potentially mobile form was studied. The soil sample was sequentially treated according to the technique that was proposed by Tessier (as described in [12]) to determine the fractions bounded with Fe, Mn-(hydr)oxides, and OM after leaching the actual mobile form.

The solutions were analyzed by a mass spectrometer with inductively coupled plasma (Perkin Elmer ELAN 9000 DRC-e, Waltham, MA, USA). Multielement calibration solutions Inorganic Ventures (Christiansburg, VA, USA) (IV-STOCK-29, IV-STOCK-21, IV-STOCK-28) were used for the instrumental calibration. Standard reference materials were used for quality control: CRM-SOIL-A (High purity standards, Charleston, SC, USA), GSO 7126-84 Bil-1, GSO 902-76 SP-2, GSO 903-76 SP-3, SDPS, SKR-1 (Irkutsk, Russia).

### 2.3. Statistical Analysis

Each determination was performed in five replicates. The precision of detection was 0.1–3% for Ni, Cu, Pb, Cd, Si, Mn, and 6–15% for other elements.

## 3. Results

### 3.1. Characteristics of Industrially Polluted Peat Soil

The pH of the peat soil was 3.7 ± 0.1, Eh-284 ± 15 mV, and the content of organic carbon was 33 ± 2%. Table 2 presents the content of total and actually mobile forms of chemical elements determined by single extraction by AAB in the peat soil. The high content of the main toxicants of the Cu/Ni smelter Cu and Ni should be noted. The table also presents data on the share of the mobile form of chemical elements in their total content. The highest values of this parameter in the PTM group were observed for industrial pollutants—Cu (54%), Pb (32%), Se and Cd (26–27%), and Ni and Sb (18–19%).

### 3.2. Effect of pH of AAB on the Content of the Actually Mobile Form of Metals

We can determine the content of AAB-soluble metal fraction at different pH values by changing the composition (and pH) of AAB. It should be noted that the acetate ion content decreases in AAB solutions at high pH values, which leads to the predominance of the cation exchange process occurring in the solution. The acetate ion content becomes higher at low pH values, and complexation becomes the primary mechanism of cation retention in the extracting solution.

**Figure 1:**
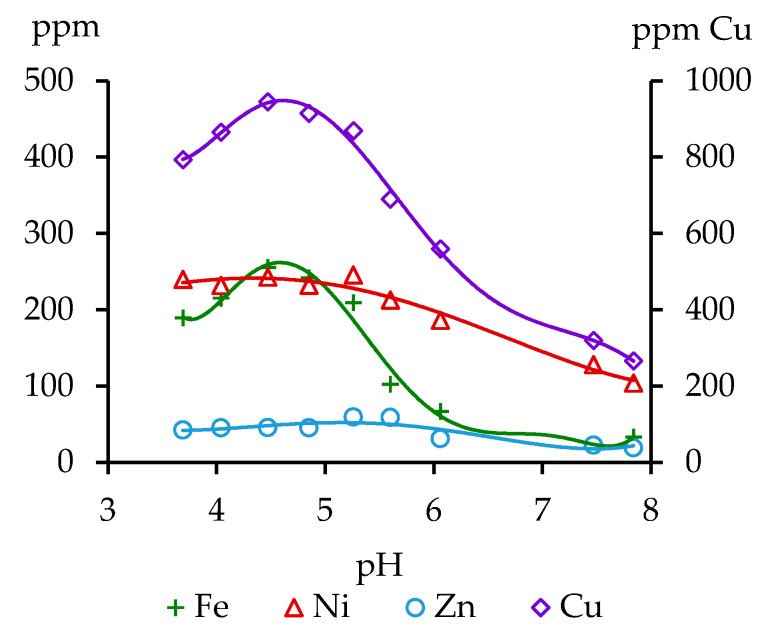
Comparison in level of the metal extraction at different pH of ammonium acetate buffer (AAB).

Figure 1 shows the metal content that was extracted by AAB at different pH values. The Ni content was the highest (230–240 ppm) at pH values less than 5.5 and it gradually decreased to 100 ppm with an increase in pH to ~8. The same monotonic dependence for Zn was observed, but the values of Zn that were extracted were much lower than those for Ni—20–50 ppm.

For Cu extraction, we observed the high maximum value at pH ~4.5; the concentrations of Cu decrease at pH value higher or lower than 4.5. This result differs from the data that were reported for soils with low content of organic carbon [2] that can be explained by the polyelectrolyte nature of the humified organic matter and the presence of various types of sorption centers in it. The organic matter more actively bounds copper when compared with nickel [29], for which a decrease in mobility in acidic environment was not detected. A similar tendency was observed for Fe. Thus, the maximum metal concentrations in the solutions were at the pH of AAB in the range of 4.5–5.5.

### 3.3. Effect of Extraction Conditions on the Fractions Extracted by Water, AAB, and 0.1 N HNO_3_

**Figure 2:**
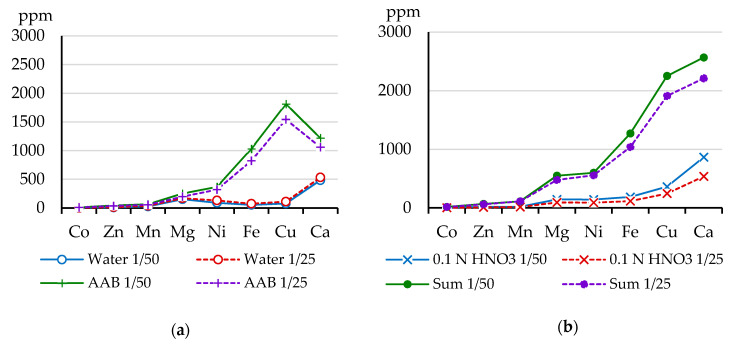
The content of element fractions after sequential treatment with solutions: (**a**) water and AAB; (**b**) 0.1 N HNO_3_ and the sum of fractions at the ratio of peat to solutions 1 g/50 mL (solid line) and 1 g/25 mL (dash line).

Figure 2 represents the content of chemical elements in fractions that were sequentially extracted by water, AAB, and 0.1 N nitric acid with the ratio between soil and solution 1:50 and 1:25, respectively. In the water-soluble fraction, the content of elements in both variants was similar for Ca and Mg or decreased with the increase of water volume for other studied metals (Figure 2a). In the fraction that was extracted by AAB, the content of dominant pollutants, Ni and Cu (as well as Fe also occurred in emissions), was 2–10 times higher than their content in water-soluble fraction. The amounts of these elements extracted by AAB and 0.1 N HNO_3_ also increased with a decrease of extractant volume (Figure 2a,b). The ratio of peat to solutions practically did not markedly affect the total amount of elements in the water-soluble, AAB-extractable, and 0.1 N HNO_3_ -extractable fractions.

**Figure 3:**
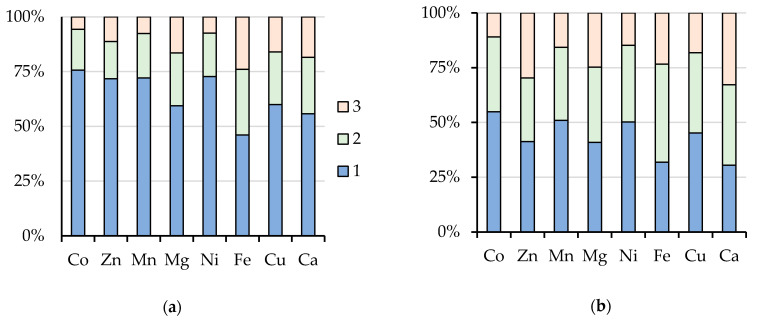
The ratio between the content of elements extracted by water (**a**) and AAB (**b**) in the 1st, 2nd, and 3rd treatment (100% is the total content of corresponding elements after three treatments).

Multiple treatments of peat soil markedly increased the total element amounts in the solution (Figure 3). In particular, the amount of elements extracted by single extraction with water was less than 50% for Fe, 55–60% for Cu, Mg, Ca, and 70–75% for Co, Zn, Mn, and Ni as compared with the content of these elements after threefold extraction (Figure 3a). Repeated treatment by AAB also affects the extraction level (Figure 3b). The amount of extracted elements was similar in the first and second treatments and decreased in the third treatment, but it remained relatively high. The share of content of Ni and Cu extracted in the third treatment was 15% and 20%, respectively.

Data on the element distribution in geochemical fractions show that triple treatment with AAB solution leads to an increase in the proportion of mobile forms in the total content of elements in the soil. Silicon was the least mobile element (10%), whereas the content of a mobile form of such PTM as Cu, Ni, Co, and Cd was 62–88% (Table 3). For these elements, the multiplicity of treatment has a considerable effect on the distribution between geochemical fractions.

**Figure 4:**
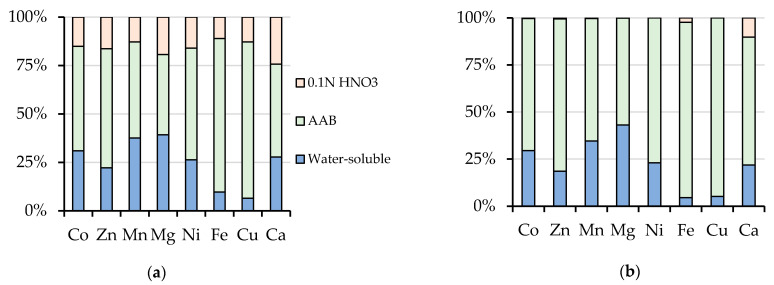
The ratio between the total content of water-soluble, AAB, and 0.1 N HNO_3_-extracted fractions of elements after a single (**a**) and sequential triple (**b**) treatment of peat with extracting solutions.

A comparison between the content of chemical elements extracted by 0.1 N HNO_3_ and AAB showed that the amount of metals extracted by 0.1 N HNO_3_ was almost the same as their total content after threefold sequential extraction by water and AAB (Figure 4). Therefore, the single treatment of peat soil by weak nitric acid instead of threefold treatment by AAB has been proposed to find the total amount of the mobile metal compounds (the sum of water-soluble and exchangeable fractions).

### 3.4. Potentially Mobile Form of Chemical Elements

Table 3 presents the results of the determination of elements in the sequential extraction procedure. The sum of element content in three fractions obtained in the series of the experiment with the single and triple treatment of AAB did not statistically differ (ANOVA-test, p < 0.1).

An increase in the proportion of the actual mobile form (water-soluble ad exchangeable fractions) after the triple treatment led to the redistribution of elements in the potentially mobile form. The percentage of components that were associated with Fe, Mn-hydroxides naturally decreased in the series with the three-fold treatment by AAB, and this tendency was observed for all of the investigated elements (Table 3). The share of this form for Cu, Ni, Co, and Cd decreased by ~20%, whereas, for Fe -7%, for Mn -15%. The change in the share of fraction bound with OM after the triple treatments was about 10% for most PTM and less than 10% for the other elements.

## 4. Discussion and Conclusions

In the first part of the study, we investigated the effect of the pH values of the AAB solution on the content of the water-soluble and exchangeable metal fractions. AAB with a pH of 7.0 is used to determine the content of mobile metal forms in most of the international protocols [17,30,31]. At the same time, the technique of extracting with AAB at pH of 4.65–4.8 is conventional in Russian soil science [12,14]. Thus, we conducted the AAB extraction of elements in the wide pH range (3.7–7.8) and studied the effect of pH on the content of metals in the industrially polluted peat soil.

First of all, the soil that was studied in this research was characterized by a high content of mobile Cu. The content of elements extracting by AAB was found to be highly dependent of the pH value for Cu and Fe. The concentration of metals in the resulted solutions was maximum at the pH of AAB within a range of 4.5–5.5. Gumbara and Sumawinata [32] suggested that the analysis of metals in soils with a high content of OM should be carry out at pH values of AAB solution proper to the peat soil in nature. Therefore, the treatment of acid peat soil by AAB with pH 4.65–4.8 is the most appropriate for determining the mobile forms of Cu and other metals.

In the second part of the study, we investigated the effect of repeated soil treatment with extractant on the concentration of metals in the resulting solution, as well as the effect of the increased volume of the extractant. The obtained results indicate the incomplete extraction of water-soluble and exchangeable fractions from highly polluted peat soil when carrying out analyses using the single treatment. This problem is known, especially for soils with a high content of OM [33]. Our results showed that a decrease in the ratio between the weight of soil sample and amount of extractant (AAB) led to a slight increase in the extraction efficiency. At the same time, repeated extraction with solutions (water and AAB) led to a marked increase in the content of chemical elements in the resulted solution. Multiple treatments at least three times increased the cost of analytical work. In this case, it is advisable to analyze the joint sample. Another way to solve this problem is to develop methods while using more active and preferably unbuffered extractants [33]. In the experiment using 0.1 N HNO_3_, we demonstrated that the fraction extracted by this solution could be, to the same degree, extracted by three-fold treatment with AAB.

In the third part of the study, we examined the effect of the full extraction of chemical elements in actually mobile form of elements (water-soluble and exchangeable) while using triple soil treatment by AAB solution on their content in potentially mobile form. Full extraction led to a redistribution of the portion of chemical elements between exchangeable fraction and fractions bounded by Fe/Mn-(hydr)oxides and OM. More stringent extraction conditions lead to the extraction of sorbed forms, the bond strength of which with the carrier phases can be described as less strong. The removal of this fraction does not require the destruction of the carrier phases according to the Tessier method. Information regarding the amount of such forms can be useful in evaluating the changes in the geochemical mobility of metals at alkaline geochemical barriers during soil remediation while using vermiculite- and serpentine rich mining waste [22].

Thus, we propose the following modification for the Tessier’s scheme of the sequential extraction of different metal fractions to estimate the geochemical mobility of metals in highly polluted peat soil: (1) three-fold treatment with distilled water; (2) single extraction with AAB; (3) extraction with 0.1 N HNO_3_; (4) extraction of fraction associated with (hydr)oxides of Fe and Mn; and, (5) extraction of fraction bound by OM. Among these fractions, fractions 1–3 are referred to the actually mobile form, fractions 4–5 to the potentially mobile form.

The method of the metal extraction with AAB solutions in wide pH range is a useful tool for studying the influence of various factors on the content of mobile components in polluted soil [2]. The dividing of the actually mobile metal form into water-soluble, extracted by the single treatment with AAB, and extracted by diluted nitric acid is necessary for understanding the processes of transformation of the most toxic PTM fractions. This study has several practical applications in the carrying out of chemical analyses of soils with high level of industrial pollution by metals. The altered procedure of metal sequential extraction allows for evaluating geochemical mobility of metals and current environmental harm of polluted soils with a high content of organic matter. Additionally, this might be applicable to remediation of soil polluted with PTM with use of materials that reduce the mobility of metals.

## Figures and Tables

**Table 1:** Ecological significance of geochemical fractions of metals released during sequential extraction (according to [9,11,12,13,14]).

Metal Fraction	Extractant	Mechanism of Extraction	Migration Forms	Mobile Forms
**Water-soluble**	H_2_O	Dissolution	Biologically available	Actual mobile
**Exchangeable**	NH_4_CH_3_COO	Ion exchange, complexation		
**Bound by Fe/Mn (hydr)oxides**	NH_2_OH∙HCl	Destruction of carrier phases (reducible conditions)	Strongly associated with carrier phases	Potentially mobile
**Bound by OM**	H_2_O_2_	Destruction of carrier phases (oxidizable conditions)		
**Residual**	HF + HClO_4_	Full autopsy	Not migrate	Strongly bound

**Table 2:** The total and mobile content of elements in peat soil, ppm.

Element	Ca	Mg	Al	Si	Fe	Mn	Pb	Zn	Cr	Cd	Co	Ni	Cu	Sb	Se
**Total**	3376	1623	11	64	26	265	19	93	132	0.38	83	2344	5955	1.15	7.3
**Mobile**	718	103	85	30	2553	15	6	4	7	0.10	3	440	3200	0.20	2
**Mobile/total, %**	21	6	1	0.05	10	5	32	5	6	26	4	19	54	18	27

**Table 3:** The content of elements in geochemical fractions.

Element	Number of Treatments	Content, ppm				Distribution, %		
		Exch.	Fe/Mn-ox.	OM	Sum	Exch.	Fe/Mn-ox.	OM
**Cu**	1	2081	2309	1917	6308	33	37	30
	3	3695	1084	1166	5945	*62*	18	20
**Ni**	1	472	340	303	1114	42	31	27
	3	795	144	172	1111	*72*	13	15
**Co**	1	17	15	7	40	43	39	18
	3	40	9	4	53	*75*	16	8
**Fe**	1	1535	10	6398	18	8	57	35
	3	2697	8242	5533	16	16	50	34
**Mn**	1	92	56	26	175	53	32	15
	3	135	31	20	186	73	17	11
**Cr**	1	11	34	26	71	15	49	36
	3	39	31	25	95	41	33	27
**Pb**	1	3	6	8	17	16	38	46
	3	5	5	7	18	29	30	41
**Cd**	1	0.70	0.26	0.07	1.03	68	25	7
	3	0.94	0.09	0.04	1.07	88	8	4
**Al**	1	517	1088	2852	4457	12	24	64
	3	971	585	2347	3904	25	15	60
**Ca**	1	1310	868	720	2898	45	30	25
	3	2613	539	643	3795	69	14	17
**Mg**	1	327	175	409	911	36	19	45
	3	471	113	446	1030	46	11	43
**Si**	1	21	916	1092	2028	1	45	54
	3	213	664	1231	2108	10	31	58
**K**	1	39	41	49	129	30	32	38
	3	48	27	70	145	33	19	48

Content of elements in the exchangeable + water-soluble fractions (Exch.), bounded by (hydr)oxides of Fe and Mn (Fe/Mn-ox.), bounded by organic matter (OM), and the sum of fractions (Sum).

## References

[1] Tokalioğlu Ş., Kartal Ş., Birol G. (2003). Application of a three-stage sequential extraction procedure for the determination of extractable metal contents in highway soils. Turk. J. Chem..

[2] Tokunaga S., Park S.W., Ulmanu M. (2005). Extraction behavior of metallic contaminants and soil constituents from contaminated soils. Environ. Technol..

[3] Antoniadis V., Levizou E., Shaheen S.M., Ok Y.S., Sebastian A., Baum C.H., Prasad M.N.V., Wenzel W.W., Rinklebe J. (2017). Trace elements in the soil-plant interface: Phytoavailability, translocation, and phytoremediation—A review. Earth-Sci. Rev..

[4] Silveira M.L., Alleoni L.R.F., O’Connor G.A., Chang A.C. (2006). Heavy metal sequential extraction methods—A modification for tropical soils. Chemosphere.

[5] Plyaskina O.V., Ladonin D.V. (2009). Heavy metal pollution of urban soils. Eur. Soil Sci..

[6] Quevauviller P., Rauret G., Griepink B. (1993). Single and sequential extraction in sediments and soils. Int. J. Environ. Anal. Chem..

[7] Rao C.R.M., Sahuquillo A., Lopez Sancher J.F. (2008). A review of the different methods applied in environmental geochemistry for single and sequential extraction of trace elements in soils and related materials. Water Air Soil Pollut..

[8] Del Castilho P., Rix I. (1993). Ammonium acetate extraction for soil heavy metal speciation; model aided soil test interpretation. Int. J. Environ. Anal. Chem..

[9] Forms of compounds of heavy metals in industrially contaminated soils. https://dlib.rsl.ru/viewer/01006646507#?page=1.

[10] Unsal Y.E., Tuzen M., Soylak M. (2014). Sequential extraction procedure for the determination of some trace elements in fertilizer samples. J. AOAC Int..

[11] Tessier A., Campbell P.G., Bisson M. (1979). Sequential extraction procedure for the speciation of particulate trace metals. Anal. Chem..

[12] Minkina T.M., Motuzova G.V., Nazarenko O.G., Kryshchenko V.S., Mandzhieva S.S. (2008). Combined approach for fractioning metal compounds in soils. Eur. Soil Sci..

[13] Minkina T.M., Motuzova G.V., Mandzhieva S.S., Nazarenko O.G., Burachevskaya M.V., Antonenko E.M. (2013). Fractional and group composition of the Mn, Cr, Ni, and Cd compounds in the soils of technogenic landscapes in the impact zone of the Novocherkassk Power Station. Eur. Soil Sci..

[14] Minkina T.M., Mandzhieva S.S., Burachevskaya M.V., Bauer T.V., Sushkova S.N. (2018). Method of determining loosely bound compounds of heavy metals in the soil. MethodsX.

[15] Sungur A., Soylak M., Ozcan H. (2014). Investigation of heavy metal mobility and availability by the BCR sequential extraction procedure: Relationship between soil properties and heavy metals availability. Chem. Speciat. Bioavailab..

[16] Sabienë N., Brazauskienë D.M., Rimmer D. (2004). Determination of heavy metal mobile forms by different extraction methods. Ekologija.

[17] Ure A.M. (1996). Single extraction schemes for soil analysis and related applications. Sci. Total Environ..

[18] Takeda A., Tsukada H., Takaku Y., Hisamatsu S., Inaba J., Nanzyo M. (2006). Extractability of major and trace elements from agricultural soils using chemical extraction methods: Application for phytoavailability assessment. Soil Sci. Plant Nutr..

[19] Normandin V., Kotuby-Amacher J., Miller R.O. (1998). Modification of the ammonium acetate extractant for the determination of exchangeable cations in calcareous soils. Commun. Soil Sci. Plant Anal..

[20] Scokart P.O., Meeus-Verdinne K., De Borger R. (1983). Mobility of heavy metals in polluted soils near zinc smelters. Water Air Soil Pollut..

[21] Misra S.G., Pande P. (1974). Evaluation of a suitable extractant for available nickel in soils. Plant Soil.

[22] Fedotova E.V., Mosendz I.A., Kremenetskaya I.P., Drogobuzhskaya S.V. (2017). Forms of deposition of copper and nickel by sungulite and thermovermiculite. Proc. Kola Sci. Cent. RAS.

[23] Kozlov M.V., Zvereva E.L. (2007). Industrial barrens: Extreme habitats created by non-ferrous metallurgy. Rev. Environ. Sci. Bio/Technol..

[24] Kashulina G.M. (2017). Extreme pollution of soils by emissions of the copper–nickel industrial complex in the Kola Peninsula. Eurasian Soil Sci..

[25] Slukovskaya M.V., Kremenetskaya I.P., Ivanova L.A., Vasilieva T.N. (2017). Remediation in conditions of an operating copper-nickel plant: Results of perennial experiment. Non-Ferr. Met..

[26] Slukovskaya M.V., Vasenev V.I., Ivashchenko K.V., Morev D.V., Drogobuzhskaya S.V., Ivanova L.A., Kremenetskaya I.P. (2019). Technosols on mining wastes in the Subarctic: Efficiency of remediation under Cu-Ni atmospheric pollution. Int. Soil Water Conserv. Res..

[27] Slukovskaya M.V., Kremenetskaya I.P., Drogobuzhskaya S.V., Ivanova L.A., Mosendz I.A., Novikov A.I. (2018). Serpentine mining wastes—Materials for soil rehabilitation in Cu-Ni polluted wastelands. Soil Sci..

[28] Gregorich E.G., Carter M.R. (2007). Soil Sampling and Methods of Analysis.

[29] Kichigin O.V. (2005). Potentiometric study of the stability of complexes of polymer chelate sorbents with ions of multivalent metals. Bull. Voronezh State Univ. Ser..

[30] Jones J.B. (1990). Universal soil extractants: Their composition and use. Commun. Soil Sci. Plant Anal..

[31] Shuman L.M., Duncan R.R. (1990). Soil exchangeable cations and aluminum measured by ammonium chloride, potassium chloride, and ammonium acetate. Commun. Soil Sci. Plant Anal..

[32] Gumbara R.H., Sumawinata B. (2019). A comparison of cation exchange capacity of organic soils determined by ammonium acetate solutions buffered at some pHs ranging between around field pH and 7.0. IOP Conf. Ser. Earth Environ. Sci..

[33] Ciesielski H., Sterckeman T., Santerne M., Willery J.P. (1997). A comparison between three methods for the determination of cation exchange capacity and exchangeable cations in soils. Agron. EDP Sci..

